# A new method for the synthesis of diamantane by hydroisomerization of binor-S on treatment with sulfuric acid

**DOI:** 10.3762/bjoc.16.205

**Published:** 2020-10-12

**Authors:** Rishat I Aminov, Ravil I Khusnutdinov

**Affiliations:** 1 Institute of Petrochemistry and Catalysis, Russian Academy of Sciences, pr. Oktyabrya 141, Ufa, 450075, Russian Federation

**Keywords:** binor-S, diamantane, hydroisomerization, sulfuric acid, tetrahydrobinor-S

## Abstract

A new method was developed for the direct synthesis of the second representative of the homologous series of diamond-like hydrocarbons, diamantane, in 65% yield by hydroisomerization of the norbornadiene dimer, *endo-endo*-heptacyclo[8.4.0.0^2,12^.0^3,8^.0^4,6^.0^5,9^.0^11,13^]tetradecane (binor-S) on treatment with concentrated sulfuric acid (98%). In the presence of H_2_SO_4_ of lower concentration (75–80%), the reaction stops after the hydrogenation step giving *endo-endo*-pentacyclo[7.3.1.1^2,5^.1^8,10^.0^3,7^]tetradecane in 68% yield with excellent selectivity (100%).

## Introduction

Among the highly diverse polycyclic and cage compounds, an important place is occupied by diamond-like compounds called diamondoids, whose lower representatives belong to the homologous series C_4_*_n_*_+6_H_4_*_n_*_+12_. Owing to the rigid structure, diamondoids typically have high thermal stability and high reactivity compared with aliphatic and alicyclic saturated hydrocarbons and show peculiar chemical behavior.

Crude oil is known to be the main natural source of diamondoids. In the oil and gas field exploration, the presence of diamondoids is used to evaluate the field maturity. Whereas the synthesis and chemical reactivity of adamantane, the first member of the diamondoid homologous series, which is produced on an industrial scale (prepared by AlBr_3_ or AlCl_3_-induced skeletal isomerization of a petrochemical monomer, hydrogenated dicyclopentadiene) [1], have been studied rather extensively, the chemical behavior of diamantane, the second member of the diamandoid homologous series, has been poorly studied. The main cause of this situation is the lack of facile methods for its synthesis.

**Scheme 1:**
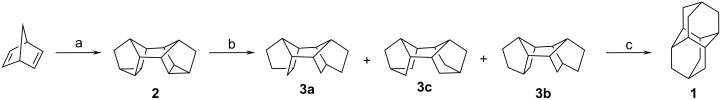
Isomerization of **3а**–**с** to diamantane (**1**). Reaction conditions: (a) CoBr_2_·2PPh_3_–BF_3_·OEt_2_, 110 °C, 12 h; (b) Pt, H_2_ (200 psi), 70 °C, 3 h; (c) superacidic catalysts or NaY–NaH.

In the literature, diamantane (**1**) is prepared by skeletal isomerization of strained С_14_Н_20_ polycyclic hydrocarbons [2–7]. In particular, the most suitable initial compounds for the preparation of diamantane are three isomeric polycyclic hydrocarbons C_14_H_20_ **3а**–**с**, which are obtained by hydrogenation of the norbornadiene dimer, heptacyclo[8.4.0.0^2,12^.0^3,8^.0^4,6^.0^5,9^.0^11,13^]tetradecane (binor-S, **2**). Binor-S is hydrogenated in the presence of a platinum catalyst (Н_2_PtCl_6_, PtO_2_) in glacial acetic acid under high pressure conditions at 70 °С and 200 psi of H_2_ [8–9]. In the presence of superacid catalysts, such as B(OSO_2_CF_3_)_3_, CF_3_SO_3_H/SbF_5_ 1:1, CF_3_SO_3_H/B(OSO_2_CF_3_)_3_ 1:1 [10], NaBH_4_/CF_3_SO_3_H [11], or zeolite Y in the NaH form (NaY) [12], hydrocarbons **3a**–**c** isomerize to diamantane in up to 99% yield (Scheme 1).

As can be seen from Scheme 1, the synthesis of diamantane (**1**) from binor-S (**2**) is a two-step process, in which the hydrogenation performed in the first step is most complex and has always been an obstacle to the generation of large amounts of diamantane. In view of the foregoing, we set ourselves the task to develop a one-pot method for the synthesis of diamantane (**1**) from binor-S (**2**).

## Results and Discussion

**Scheme 2:**
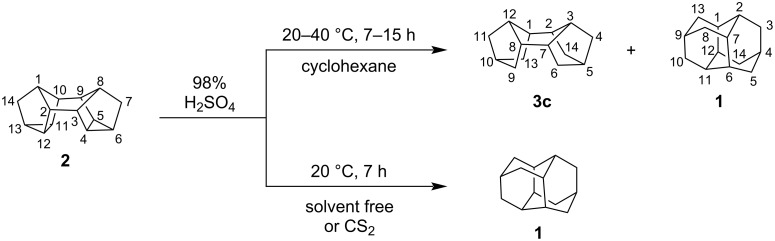
Isomerization of binor-S (**2**) to diamantane (**1**).

In this study, we developed a new method for the synthesis of pentacyclo[7.3.1.1^4,12^.0^2,7^.0^6,11^]tetradecane (diamantane, **1**) by skeletal hydroisomerization of *endo-endo*-heptacyclo[8.4.0.0^2,12^.0^3,8^.0^4,6^.0^5,9^.0^11,13^]tetradecane (binor-S, **2**) on treatment with sulfuric acid (Scheme 2).

The reaction selectivity and the yield of diamantane (**1**) considerably depend on the reaction conditions and the solvent nature. Indeed, at 20–40 °C, hydroizomerization of binor-S (**2**) in cyclohexane in the presence of 98% sulfuric acid ([**2**]/[H_2_SO_4_] = 1:10–50) during 7–15 h affords a mixture of *endo-endo*-pentacyclo[7.3.1.1^2,5^.1^8,10^]tetradecane (tetrahydrobinor-S, **3c**) and diamantane (**1**) (Table 1). An increase in the sulfuric acid ratio to binor-S (**2**) ([**2**]/[H_2_SO_4_] = 1:20–50) and rising the temperature to 40 °С lead to decreased product yield due to resinification. When the H_2_SO_4_ ratio to binor-S (**2**) is 1:5, the conversion of compound **2** decreases to 10%. On the other hand, when the reactions are carried out in CS_2_ or without any solvent, the selectivity to diamantane (**1**) increases to 100%, with the maximum yield being 65% (Table 1, entry 12). A portion of binor-S (**2**) is converted to resinous products. When the reaction was ultrasonically assisted, the reaction time decreased to 2 h with the yield of diamantane (**1**) being retained (62%).

**Table 1:** Hydroisomerization of binor-S (**2**) in the presence of sulfuric acid.

entry	ratio	solvent	temp. [°C]	time [h]	product ratio [%]^a^		
					
	[**2**]/[H_2_SO_4_]				**2**	**3**	**1**

1	1:50	cyclohexane	40	7	2	10	23
2	1:50	cyclohexane	20	7	3	26	10
3	1:20	cyclohexane	40	7	3	52	28
4	1:20	cyclohexane	20	7	12	46	22
5	1:20	cyclohexane	20	15	–	55	31
6	1:10	cyclohexane	20	7	22	41	36
7	1:10	cyclohexane	20	15	16	47	34
8	1:5	cyclohexane	40	15	56	31	2
9	1:20	carbon disulfide	20	7	21	–	36
10	1:20	carbon disulfide	20	15	15	–	44
11	1:10	carbon disulfide	20	7	24	–	52
12	1:10	carbon disulfide	20	15	–	–	65
13	1:5	carbon disulfide	40	7	78	–	10
14	1:5	carbon disulfide	20	7	90	–	–
15	1:10	–	20	7	–	–	8
16^b^	1:10	cyclohexane	20	2	9	64	26
17^b^	1:10	carbon disulfide	20	2	–	18	62
18^b^	1:10	–	20	2	–	–	6

^a^Determined by GC using C_12_H_26_ as the internal standard. ^b^The reaction was conducted under ultrasonic irradiation.

In order to answer the question of what is the hydrogen source in the hydroisomerization of binor-S (С_14_H_16_, **2**) containing 4 hydrogen atoms less than diamantane (С_14_H_20_, **1**), we carried out a series of control experiments using deuterated sulfuric acid (98%) in cyclohexane (С_6_H_12_, experiment A), in deuterated cyclohexane (C_6_D_12_, experiment B), or in carbon disulfide (CS_2_, experiment C).

In experiment А, the major isomer **1-D_2_**, which is formed upon hydroisomerization of binor-S (**2**), contains two deuterium atoms. Two more hydrogen atoms are probably provided by cyclohexane. Unexpectedly, the reaction also gave undeuterated diamantane (**1**), which may be due to deuterium exchange with hydrogen of cyclohexane under the action of D_2_SO_4_.

The major product **1-D_3_**, which is formed in experiment B with D_2_SO_4_ in C_6_D_12_ contains three deuterium atoms. The expected isomer with four deuterium atoms is formed in a minor amount. Evidently, binor-S (**2**) acts as the hydrogen source for the isomer C_14_H_17_D_3_, **1-D_3_**. Our attempt to carry out the deuteration of diamantane (**1**) with D_2_SO_4_ in carbon disulfide for 7 h at 20 °C was unsuccessful. Evidently, the deuterium exchange, resulting in the formation of diamantanes **1-D_7_** and **1-D_8_** containing 7 and 8 deuterium atoms, occurs at the hydroisomerization step (experiment C).

**Scheme 3:**
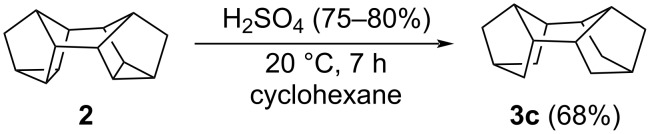
Selective synthesis of tetrahydrobinor-S (**3c**) from binor-S (**2**).

As shown by further studies, when the sulfuric acid concentration decreases to 75–80%, the reaction stops at the intermediate step giving *endo-endo*-pentacyclo[7.3.1.1^2,5^.1^8,10^.0^3,7^]tetradecane (tetrahydrobinor-S, **3с**; Scheme 3). It should be emphasized that the reaction selectively gives only one of the possible isomers, hydrocarbon **3с**, which is confirmed by ^1^H and ^13^C NMR spectral data. The ^13^C NMR spectrum of compound **3с** shows five characteristic carbon signals at 33.44, 35.64, 37.84, 38.30, and 40.49 ppm, coinciding with the reported values [13]. Since 75–80% H_2_SO_4_ contains 20–25% water, the participation of water as a hydrogen source in the reaction cannot be ruled out either.

**Scheme 4:**
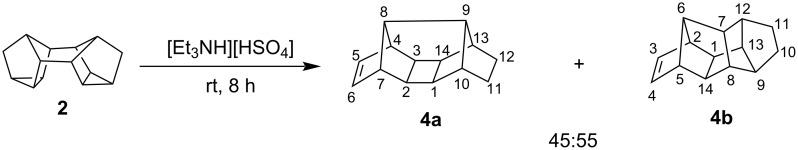
Isomerization of binor-S (**2**) to hydrocarbons **4а** and **b**.

Attempts to perform hydroisomerization of binor-S (**2**) to diamantane (**1**) on treatment with nitric or orthophosphoric acid were unsuccessful, with the starting binor-S (**2**) being recovered unchanged. The reaction of hydrocarbon **2** with hydrochloric acid proceeds with the addition of HCl to the cyclopropane ring and results in the formation of a mixture of mono- and dichloro derivatives, the synthesis of which has been reported [13–14]. When sulfuric acid is replaced by an ionic liquid prepared from triethylamine and sulfuric acid [15], the reaction follows a different route: Starting binor-S (**2**) is converted to two isomeric hexacyclic hydrocarbons, hexacyclo[8.4.0.0^2,7^.0^3,14^.0^4,8^.0^9,13^]tetradec-5-ene (**4а**) and hexacyclo[6.6.0.0.^2,6^.0^5,14^.0^7,12^.0^9,13^]tetradec-3-ene (**4b**), which are important precursors for the synthesis of triamantane [10–11,16–24] (Scheme 4).

## Conclusion

Thus, we developed a new one-pot method for the synthesis of diamantane (**1**) by hydroisomerization of binor-S (**2**) on treatment with concentrated sulfuric acid (98%) in carbon disulfide or cyclohexane. It was found that both, sulfuric acid and cyclohexane can serve as the main hydrogen sources. In the presence of H_2_SO_4_ with a lower concentration (75–80%), the reaction stops at the step of formation of *endo-endo*-pentacyclo[7.3.1.1^2,5^.1^8,10^.0^3,7^]tetradecane (**3c**) in 68% yield.

## Experimental

**General procedures and materials: **^1^H and ^13^С NMR spectra were measured on a Bruker Avance-III 400 Ascend instrument (400 MHz for ^1^Н and 100 MHz for ^13^С in CDCl_3_). Mass spectra were run on a Shimadzu GCMS-QP2010Plus mass spectrometer (SPB-5 capillary column, 30 m × 0.25 mm, helium as the carrier gas, temperature programming from 40 to 300 °С at 8 °C/min, evaporation temperature of 280 °С, ion source temperature of 200 °С, and ionization energy of 70 eV). The elemental composition of the samples was determined on a Carlo Erba 1106 elemental analyzer. The course of the reaction and the purity of the products were monitored by gas liquid chromatography on a Shimadzu GC-9A, GC-2014 instrument [2 m × 3 mm column, SE-30 silicone (5%) on Chromaton N-AW-HMDS as the stationary phase, temperature programming from 50 to 270 °С at 8 °C/min, helium as the carrier gas (47 mL/min)].

The sonication was carried out with an ultrasound generator IL10–0.63 (INLAB LTD) for 180 min at a frequency of 22 kHz with a submerged 15 mm diameter titanium horn, with output power 150 W. The reactions were carried out in a 100 × 35 mm glass reactor equipped with a jacket to maintain the required temperature (20 °C).

**Preparation of diamantane:** Heptacyclo[8.4.0.0^2,12^.0^3,8^.0^4,6^.0^5,9^. 0^11,13^]tetradecane (**2**, 0.368 g, 2 mmol) and the solvent were charged into a glass reactor (*V* = 100 mL). Then, concentrated (98%) sulfuric acid (1.96 g, 20 mmol) was added in portions with vigorous stirring. When the whole amount of H_2_SO_4_ has been added, the reaction mixture was stirred at 20 °С for 15 h. After completion of the reaction, 10% NaOH was added to the reaction mixture, the organic phase was separated, and filtered through a silica gel layer (with petroleum ether as the eluent). The solvent was distilled off and the residue was recrystallized from a 1:1 ethyl acetate/cyclohexane mixture. The characteristic data and graphical spectra of diamantane are almost identical with the literature data [25].

**Preparation of *endo*-*endo*-pentacyclo[7.3.1.1^2,5^.1^8,10^.0^3,7^]tetradecane (tetrahydrobinor-S, 3c):** Heptacyclo[8.4.0.0^2,12^.0^3,8^.0^4,6^.0^5,9^. 0^11,13^]tetradecane (**2**, 0.368 g, 2 mmol) was charged into a glass reactor (*V* = 100 mL) and dissolved in cyclohexane (10 mL). Then, 75–80% sulfuric acid (1.96 g, 20 mmol) was added in portions with vigorous stirring. When the whole amount of H_2_SO_4_ has been added, the reaction mixture was stirred at 20 °С for 7 h. After completion of the reaction, 10% NaOH was added to the reaction mixture, the organic part was separated, and filtered through a silica gel layer (with petroleum ether as the eluent). The solvent was distilled off and the residue was recrystallized from a 1:1 ethyl acetate/cyclohexane mixture. Colorless crystals; 68% yield; mp 104–106 °C; ^1^H NMR (400 MHz, CDCl_3_) δ 0.95–0.98 (m, 4H), 1.38 (s, 8H), 1.66–1.71 (m, 4H), 1.99–2.01 (m, 2H), 2.12–2.16 (m, 2H); ^13^С NMR (100 MHz, CDCl_3_) δ 33.42 (С^6^, С^9^, C^13^, C^14^), 35.63 (С^1^, С^2^, C^7^, C^8^), 37.82 (С^5^, С^10^), 38.27(С^3^, С^12^), 40.47 (С^4^, С^11^); EIMS (70 eV, *m*/*z*): 188 [M]^+^ (100), 187 (35), 159 (24), 145 (23), 131 (38), 117 (25), 105 (39), 91(82), 79 (57), 67 (29), 41 (47) %; Anal. calcd for C_14_H_20_: С, 89.29; H, 10.71; found: С, 89.14; H, 10.86*.*

**Preparation of hexacyclo[8.4.0.0^2,7^.0^3,14^.0^4,8^.0^9,13^]tetradec-5-ene (4a) and hexacyclo[6.6.0.0.^2,6^.0^5,14^.0^7,12^.0^9,13^]tetradec-3-ene (4b):** Heptacyclo[8.4.0.0^2,12^.0^3,8^.0^4,6^.0^5,9^. 0^11,13^]tetradecane (**2**, 0.368 g, 2 mmol) was charged into a glass reactor (*V* = 100 mL) and dissolved in cyclohexane. Then, [Et_3_NH]^+^[HSO_4_]^−^ (1.99 g, 10 mmol) was added and the reaction mixture was stirred at 40 °С for 8 h. Then the reactor was cooled to room temperature, the reaction mixture extracted with petroleum ether, and filtered through a silica gel layer (with petroleum ether as the eluent). Hexacyclo[8.4.0.0^2,7^.0^3,14^.0^4,8^.0^9,13^]tetradec-5-ene (**4а**) and hexacyclo[6.6.0.0.^2,6^.0^5,14^.0^7,12^.0^9,13^]tetradec-3-ene (**4b**) (45:55). Colorless oil; 78% yield; **4a**: ^1^H NMR (400 MHz, CDCl_3_) δ 1.04 (d, *J =* 7.2 Hz, 2H), 1.41 (d, *J =* 7.6 Hz, 2H), 1.95 (s, 2H), 2.09 (d, *J =* 7.2 Hz, 4H), 2.21 (d, *J =* 7.2 Hz, 2H) 2.56 (s, 2H), 5.87 (s, 2H); ^13^С NMR (100 MHz, CDCl_3_) δ 26.27 (C^11^, C^12^), 34.62 (C^10^, C^13^), 36.34 (C^1^, C^14^), 37.27 (C^2^, C^3^), 40.68 (C^4^, C^7^), 44.68 (C^8^), 52.88 (C^9^), 134.82 (C^5^, C^6^); EIMS (70 eV, *m*/*z*): 184 [M]^+^ (44), 169 (14), 155 (16), 142 (34), 117 (100), 115 (37), 105 (22), 91 (73), 80 (38), 65 (17), 41 (21) %; **4b**: ^1^H NMR (400 MHz, CDCl_3_) δ 1.19–1.24 (m, 1H), 1.31–1.36 (m, 1H), 1.48 (s, 1H), 1.56–1.59 (m, 2H), 1.71 (t, *J =* 6 Hz, 1H) 2.03–2.06 (m, 3H), 2.15–2.17 (m, 2H), 2.22 (s, 1H), 2.52 (s, 2H), 2.59 (s, 1H), 5.96–5.98 (m, 1H); ^13^С NMR (100 MHz, CDCl_3_) δ 24.08 (C^10^), 27.16 (C^11^), 40.52 (C^1^), 40.93 (C^12^), 42.30 (C^14^), 45.66 (C^9^), 47.38 (C^2^), 47.94 (C^13^), 48.61 (C^7^), 50.20 (C^8^), 54.09 (C^5^), 60.05 (C^6^), 133.69 (C^4^), 133.75 (C^3^); EIMS (70 eV, *m*/*z*): 184 [M]^+^ (40), 169 (21), 155 (45), 141 (45), 129 (51), 117 (100), 115 (53), 91 (88), 78 (43), 65 (21), 41 (20) %.

## Supporting Information

File 1 Experimental procedures, NMR, and mass spectral data.
