# Supplementary material for: A new method for the synthesis of diamantane by hydroisomerization of binor-S on treatment with sulfuric acid
Source: Beilstein J Org Chem. 2020 Oct 12;16:2534–9. doi: 10.3762/bjoc.16.205 (PMC7588729; doi:10.3762/bjoc.16.205)
Supplement: File 1 — Experimental procedures, NMR, and mass spectral data. [file Beilstein_J_Org_Chem-16-2534-s001.pdf]

BEILSTEIN JOURNAL OF ORGANIC CHEMISTRY

Supporting Information

for

A new method for the synthesis of diamantane by hydroisomerization of binor-S on treatment with sulfuric acid

Rishat I. Aminov and Ravil I. Khusnutdinov

Beilstein J. Org. Chem. **2020,** *16,* 2534–2539. doi:10.3762/bjoc.16.205

Experimental procedures, NMR, and mass spectral data

License and Terms: This is a supporting information file under the terms of the Creative Commons Attribution License (https://creativecommons.org/licenses/by/4.0). Please note that the reuse, redistribution and reproduction in particular requires that the authors and source are credited.
The license is subject to the *Beilstein Journal of Organic Chemistry* terms and conditions: (https://www.beilstein-journals.org/bjoc)

The graphical spectra of diamantane are identical to the literature data [Aminov, R. I., Akshieva, A. N., Khusnutdinov, R. I. *Catal. Commun*. **2019**, 130, 105756].

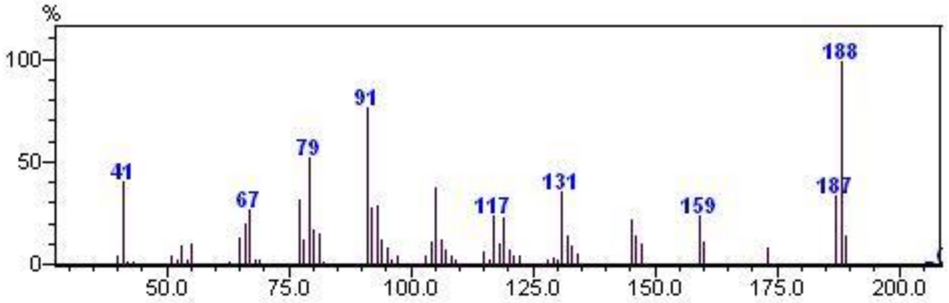

Figure S1: Mass spectrum of tetrahydrobinor-S (**3c**).

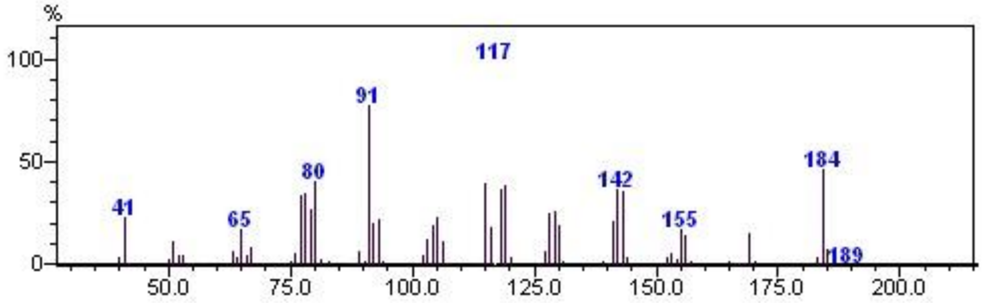

Figure S2: Mass spectrum of hexacyclo[8.4.0.0^{2,7}.0^{3,14}.0^{4,8}.0^{9,13}]tetradec-5-ene (**4a**).

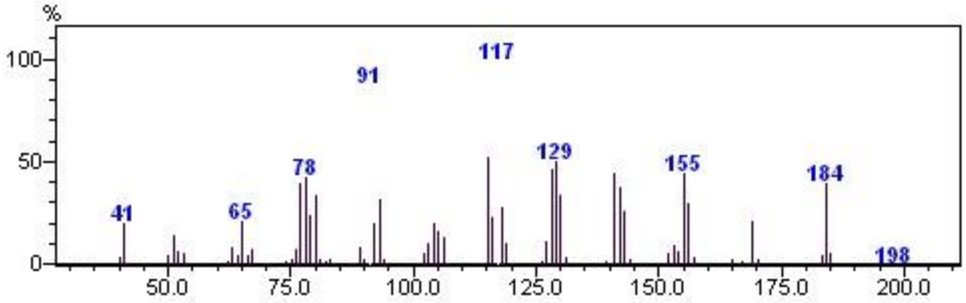

Figure S3: Mass spectrum of hexacyclo[6.6.0.0.^{2,6}.0^{5,14}.0^{7,12}.0^{9,13}]tetradec-3-ene (**4b**)

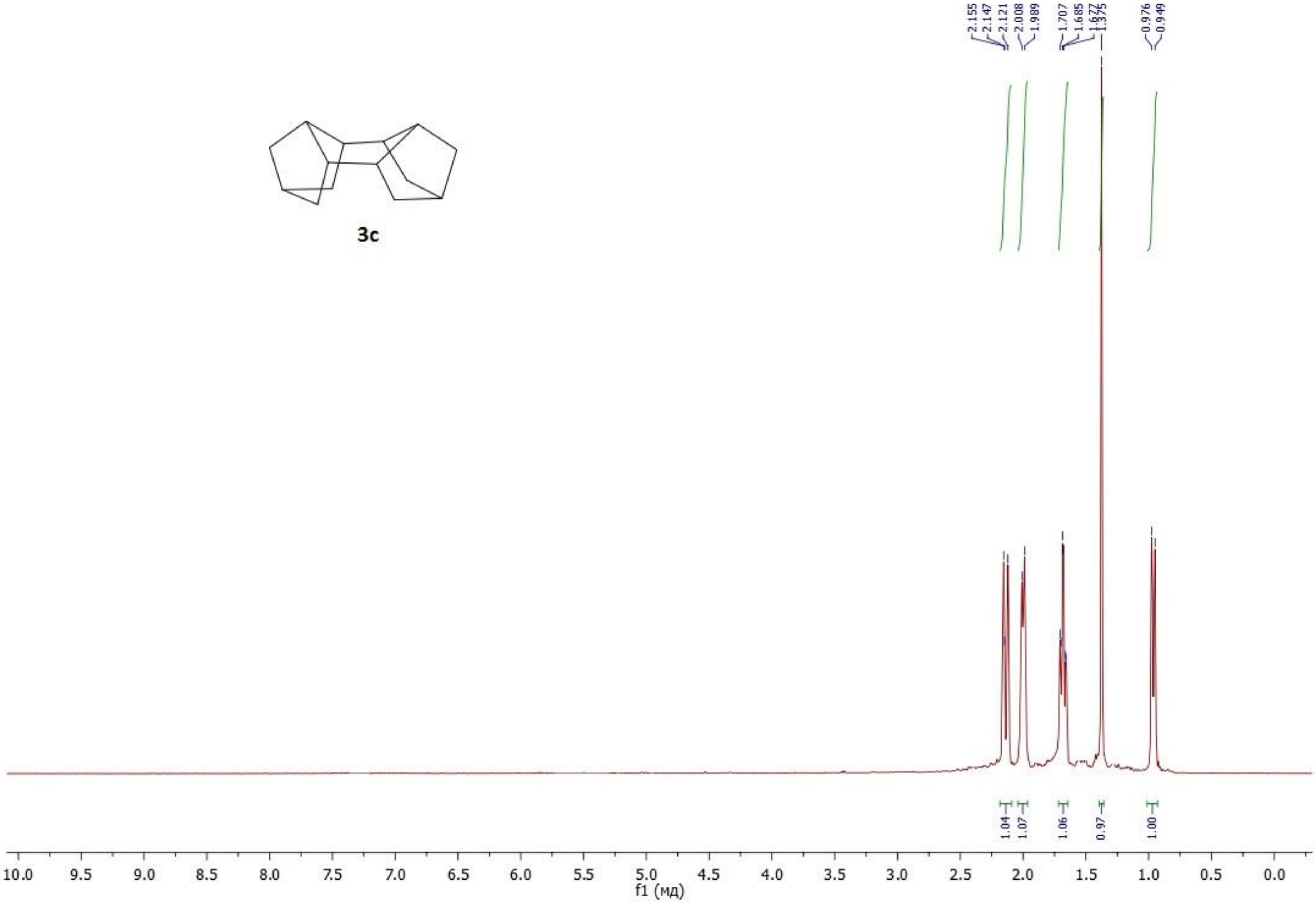

Figure S4: ^{1}H NMR spectrum of tetrahydrobinor-S (**3c**) in $CDCl_3$.

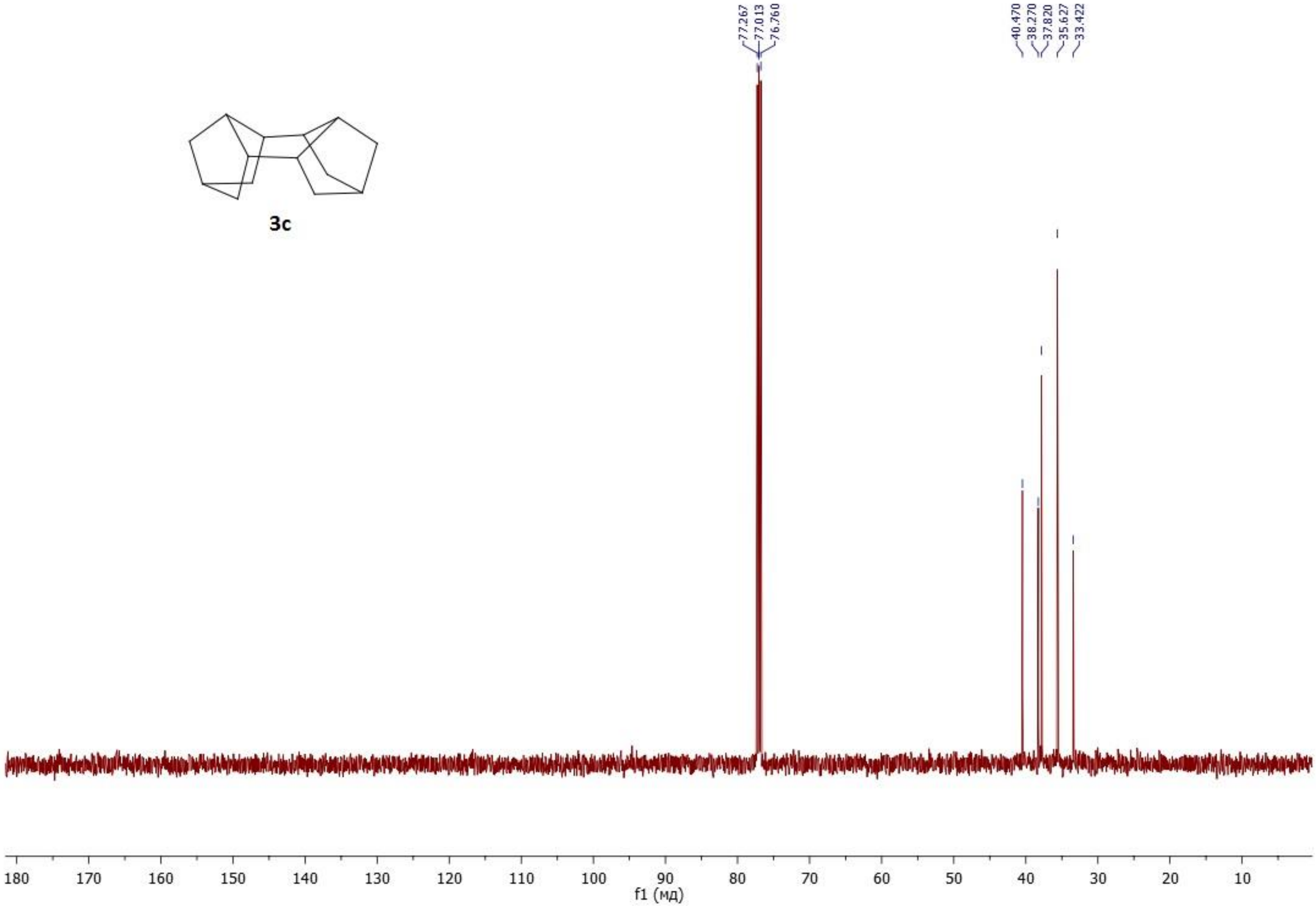

Figure S5: ^{13}C NMR spectrum of tetrahydrobinor-S (**3c**) in $CDCl_3$.

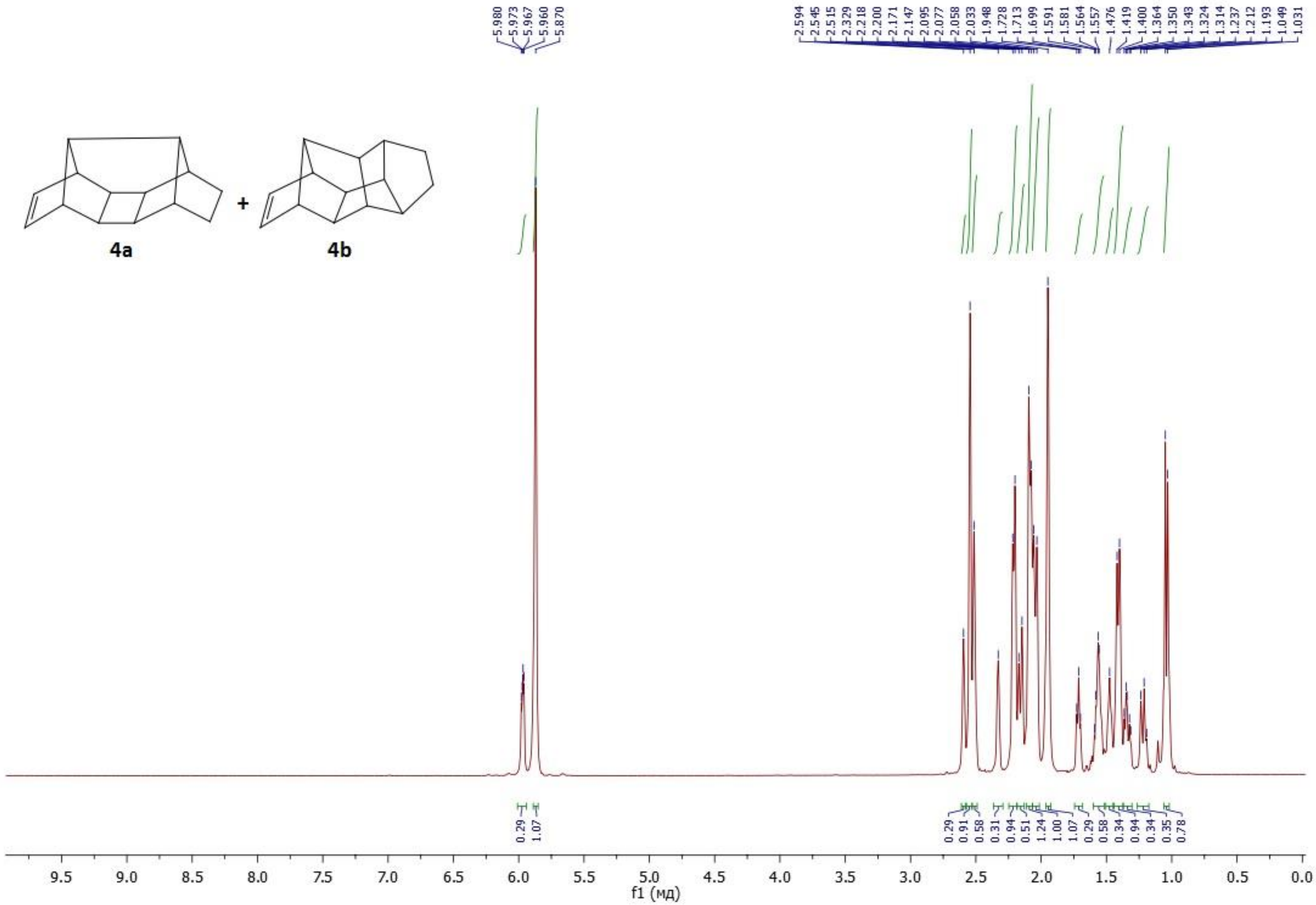

Figure S6: ^{1}H NMR spectrum of mixture hexacyclo[8.4.0.0^{2,7}.0^{3,14}.0^{4,8}.0^{9,13}]tetradec-5-ene (**4a**) and hexacyclo[6.6.0.0.^{2,6}.0^{5,14}.0^{7,12}.0^{9,13}]tetradec-3-ene (**4b**) in $CDCl_3$.

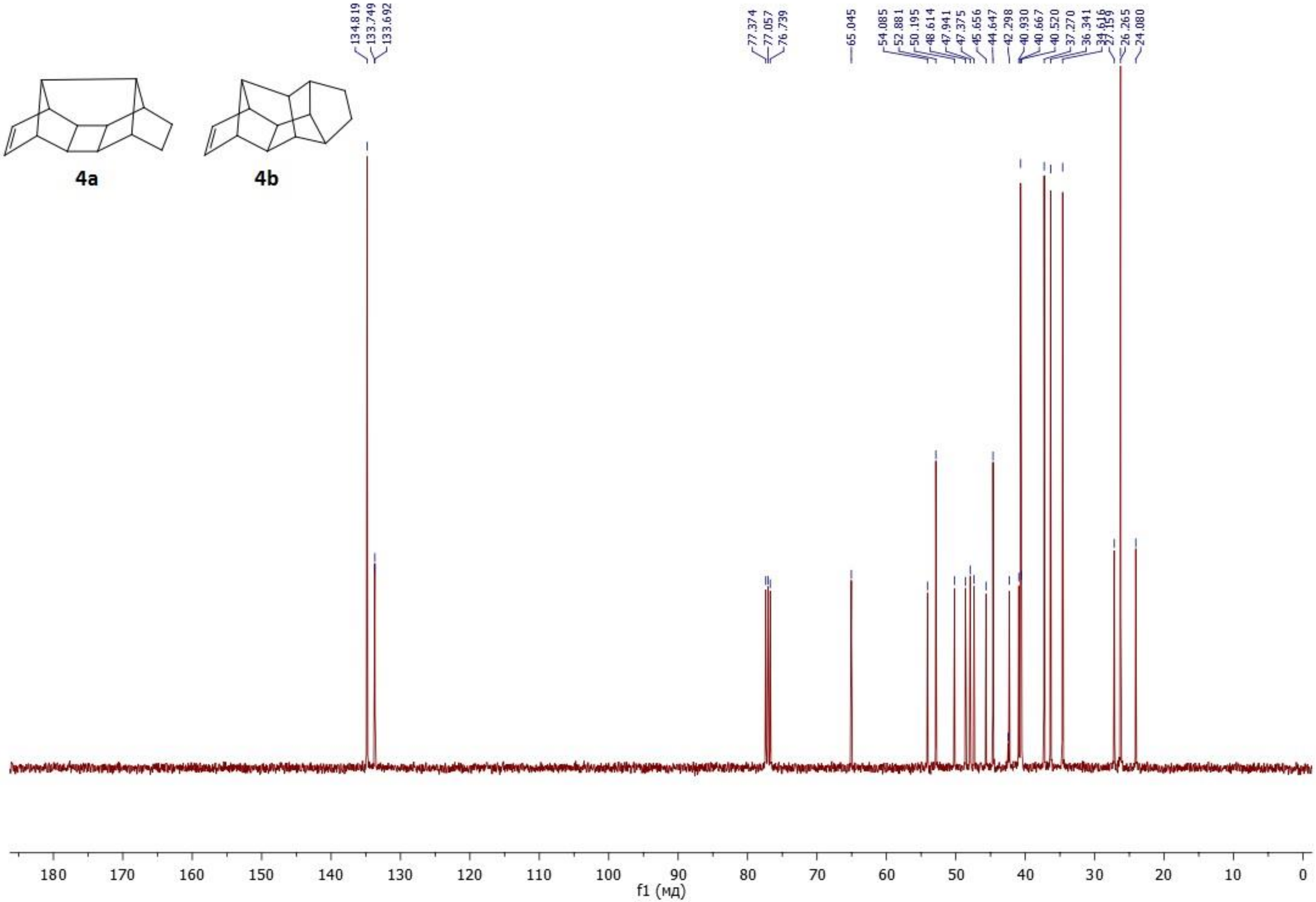

Figure S7: ^{13}C NMR spectrum of mixture hexacyclo[8.4.0.0^{2,7}.0^{3,14}.0^{4,8}.0^{9,13}]tetradec-5-ene (**4a**) and hexacyclo[6.6.0.0.^{2,6}.0^{5,14}.0^{7,12}.0^{9,13}]tetradec-3-ene (**4b**) in $CDCl_3$.
